# Pharmacological Triggers of Takotsubo Cardiomyopathy: An Updated Review of Evidence and Recommendations

**DOI:** 10.2174/011573403X273613240125072754

**Published:** 2024-02-01

**Authors:** S. Arunkumar, K. Jegaverrapandi

**Affiliations:** 1 J.K.K.Nattraja College of Pharmacy, Kumarapalayam, Namakkal district, Tamilnadu, India;; 2 Department of Pharmacy Practice, J.K.K.Nattraja College of Pharmacy, Kumarapalayam, Namakkal district, Tamilnadu, India

**Keywords:** Drug triggers, takotsubo myocardiopathy, iatrogenic, catecholamine surge, drug-induced TCM (DITC), pharmacovigilance

## Abstract

**Background:**

Previous publications in 2011, 2016, and 2022 have presented lists of drugs associated with takotsubo cardiomyopathy (TCM). This review aims to provide updated drug lists that have been reported as potential causes of TCM.

**Methods:**

Following the same methodology employed in previous reviews, a detailed investigation was carried out in the PubMed/Medline database from June 2022 to July 2023 to identify drug-induced TCM (DITC) case reports. Various search terms related to the drug-induced transient left ventricular ballooning syndrome, ampulla cardiomyopathy, apical ballooning syndrome, drug-induced broken heart syndrome, drug triggered takotsubo cardiomyopathy, tako-tsubo cardiomyopathy, and iatrogenic takotsubo cardiomyopathy were utilized. Filters for full-text availability, case reports, human studies, and English language were applied. Articles reporting drugs associated with TCM development were included in the analysis.

**Results:**

Foremost 192 case reports were initially identified, with 75 drugs meeting the inclusion criteria after a thorough review. The latest revision identified seven drugs that might lead to TCM, with four drugs (57.14%) already reported in previous reviews and three drugs (42.86%) newly identified. Consequently, the updated drug list potentially triggering TCM in 2023 comprises a sum of 75 drugs.

**Conclusion:**

The recent 75 drugs provided additional evidence linking to TCM development. The updated list predominantly includes drugs that induce sympathetic overstimulation, although some drugs on the list have unclear associations with sympathetic nervous system activation.

## 1 INTRODUCTION

Takotsubo cardiomyopathy (TCM) is a syndrome characterized by left ventricle ballooning without significant coronary artery stenosis, which closely mimics myocardial infarction. The term Takotsubo originated in Japan and refers to the distinctive appearance of the left ventricle, which resembles an octopus trap. Clinically, TCM is similar to acute myocardial infarction, although it occurs without obstructive coronary artery disease or plaque rupture. TCM is derived from the pattern of wall motion abnormalities in all four regions, including apical ballooning type, focal wall motion pattern, mid-ventricular wall motion pattern, and basal wall motion pattern. These conditions share the same morphologic substrate known as contraction band necrosis, which is characterized by localized hypercontraction and the breakdown of a small cluster of myocardial cells. The symptoms of TCM include dense eosinophilic transverse bands with hypercontracted sarcomeres and an inflammatory response consisting of mononuclear cells within the interstitial space. Drug-induced Takotsubo Cardiomyopathy (DITC) can be distinguished from typical TCM by a clear association with drug exposure and does not exist with physical or emotional stressors. The specific drugs implicated in DITC may vary and can include medications that imitate the acts of catecholamines or affect sympathetic nervous system activity. Various hypotheses exist about TCM pathophysiology, yet the most likely cause is a catecholamine spike. Stress-related events trigger the release of catecholamines, which leads to direct toxicity to the myocardium and impairment of intracellular calcium handling. Dysregulated β-adrenergic receptor signaling and altered calcium kinetics contribute to myocardial stunning and contractile dysfunction, as observed in TCM. Emotional and physical stressors typically precipitate it, and certain drugs mimic the characteristics of acute coronary syndrome in patients, such as chemotherapy, non-chemotherapy vasoconstrictors, psychiatry, opioid withdrawal adrenergic drugs, cocaine, amphetamine, methamphetamine, and exogenous catecholamine. The COVID-19 pandemic has highlighted the impact of societal stress on the incidence of Takotsubo Syndrome (TTS), where factors such as financial stress, psychological trepidation, social isolation, and financial stress associated with the pandemic have resulted in a significant increase (3-4 times) in the occurrence of TTS compared to the pre-pandemic period. Mitral valve regurgitation, pulmonary edema, cardiogenic shock, LVOT obstruction, ventricular thrombus, systemic embolization, AV block, asystole, QT prolongation, atrial fibrillation, torsades de pointes, interventricular septum rupture, and ventricular free wall rupture are common complications of Takotsubo syndrome. This systematic review aims to comprehensively analyze published case reports focusing on drug-induced takotsubo cardiomyopathy (DITC). The primary objective of this review is to summarize the drugs and related case studies that have been identified as potential triggers of drug-induced TTC. The first review of drug-induced takotsubo cardiomyopathy was a 2011 publication, where 20 drugs were identified as “possibly linked” to TCM. Most medications have a direct or indirect sympathetic nervous system. An update was published in 2016, which identified 37 new drugs compared to the 2011 review, and in 2022, which identified 18 new drugs, showing that it is necessary to continuously update this topic. Therefore, this review sets out to accomplish three key objectives: 1) To systematically review and identify case reports documenting DITC, and 2) to update the 2011, 2016, and 2022 lists of drugs potentially triggering TCM [1-5].

## 2 MATERIALS AND METHODS

**Fig. (1):**
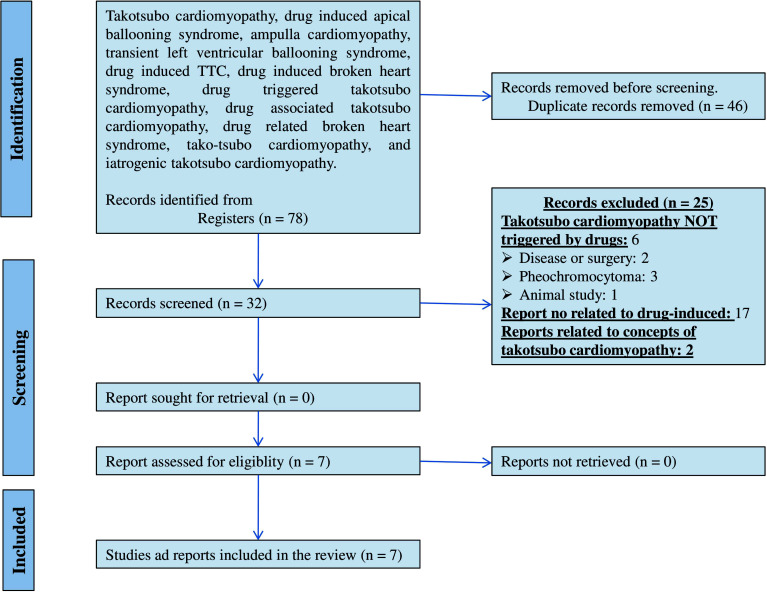
Preferred Reporting Items for Systemic Review and Meta-Analysis (PRISMA) [9] schematic representation for the systematic review of drugs that may induce TCM.

The method of this review was similar to that used in the 2011, 2015, and 2022 [6-8]. Published DITC case reports were recognized through a search using Medline/PubMed from June 2022 to July 2023. Search terms included: Drug-induced transient left ventricular ballooning syndrome (TLVBS), ampulla cardiomyopathy, apical ballooning syndrome, drug-induced TTC (DITC), drug-induced broken heart syndrome, drug-triggered takotsubo cardiomyopathy, takotsubo cardiomyopathy, and iatrogenic takotsubo cardiomyopathy. Filters for full text, case reports, humans, english, and spanish were applied. Articles were read by two authors, and any discrepancies were resolved by the third author. Additionally, the reference lists of the discovered items were thoroughly examined to identify any supplementary publications relevant to the review. The data was collected in a table that included information concerning: (a) patient age and sex, (b) the drug, route of administration, and dose used, and, (c) the disease/condition for which the drug was used, including additional clinical information (duration, prognosis, treatment, or duration of symptoms of TCM) (Fig. **1**).

## 3 RESULTS

Based on a search in the PubMed/Medline database, 78 articles were retrieved; however, 46 were repeated. The outcome is that 32 published articles were reviewed in detail (Table **1**). From the 32 screened articles, 26 were excluded. For example, 17 TCM cases were not triggered by drugs (2 were triggered by physical or emotional stress, 5 by diseases and surgery). 2 articles were specifically focused on DITC. As a result, a total of 7 papers were selected as case reports of pharmaceuticals that as potential TCM triggers. Regarding the information of these 7 patients, the average age was 45- 64 years and the range was 0.5 to 80 years; 5 (83.4%) were women, and 4 (57.14%) were above 50 years old (Table **2**). In the 7 case reports, 7 DITC were identified (Tables **2** and **3**). The drugs were: Flecainide (n =1, 14.28%), anagrelide (n =1, 14.28%), dobutamine (n =1, 14.28%), metoprolol (n =1, 14.28%), clonidine (n =1, 14.28%), tofacitinib (n =1, 14.28%), atezolizumab (n =1, 14.28%). In this group of 7 drugs, 13 (83.3%) were not identified in the 2011, 2016, and 2022 reviews. Dobutamine, the drug in question, was consistently listed in the reviews conducted in 2011, 2016, and 2023. Flecainide was common between 2015 and 2023, and Metoprolol was common between 2011 and 2023. As an outcome, the 2023 revision of the drug list linked to TCM encompasses 75 potential triggers.

## 4 DISCUSSION

In the present update of 2011 [6], 2015 [7], and 2022 [8] reviews, 7 drugs have been identified as potential TCM triggers, 4 (57.14%) have been already reported, and 3 (42.86%) were different from both previous reports. Overall, the 2023 updated compilation of medications that could potentially act as triggers for TCM consists of 75 drugs in which 34 (45.34%) cases are directed or indirectly mimic the action of the sympathetic nervous system. This list proved valuable for healthcare professionals when managing probable cases of DITC sympathetic nervous system. This list proved valuable for healthcare professionals when managing probable cases of DITC.

In total, 195 case reports described have direct sympathetic effects, thus of epinephrine (n = 32, 16.67%), norepinephrine (n = 10, 5.2%), and dobutamine (n = 20, 10.4%) were drugs identified. These findings align with the prevailing hypothesis regarding TCM development. Epinephrine was demonstrated to have the potential to induce TCM through various administration routes, including submucosal injection [90, 91], intravenous infusion [92, 93], subcutaneous injection [94, 95], nebulized inhalation [96], intramuscular injection [97-99] and intra-nasal administration [100]. In the three previous reviews, epinephrine emerged as the most frequently identified trigger, accounting for 24.65% in 2011 [6], 50.68% in 2016 [7], and 20.54% in 2022 [8], the current update, 4.1% among the total cases analyzed in each review. Increased catecholamine levels, particularly epinephrine and norepinephrine, were implicated in TTC. The excessive release of these stress hormones was believed to contribute to catecholamine-induced myocardial stunning, resulting in contractile dysfunction and exacerbation of the syndrome. Histologic findings from animal studies and autopsies of Takotsubo patients supported the concept of direct cellular toxicity, characterized by myofibrillar degeneration, leukocyte infiltration, and contraction-induced muscle tissue. *In vitro* studies on cultured cardiomyocytes demonstrated that epinephrine toxicity could directly disrupt calcium homeostasis, leading to elevated cyclic AMP and calcium levels. Consequently, this triggered free oxygen radicals production, activation of stress response genes, and apoptosis in certain cells. The compromised sarcolemma facilitated an increased influx of calcium, leading to sarcomere supercontraction, cellular disorganization, and subsequent death of myocardial cells. This process influences abnormalities of wall motion in TTC patients [101, 102].

Dobutamine, a synthetic catecholamine with selective stimulation of b1 receptors, exerted a positive inotropic effect by enhancing myocardial contractility without significantly affecting blood pressure. In certain patients, the dobutamine administration during DSE appeared to have induced TTC even if there were no emotional or stress stimuli. The overstimulation of apical adrenoceptors by dobutamine potentially exacerbated hyperdynamic basal systolic function, led to the emergence of a simulated gradient in the LVOT and added stress on the myocardium. Excessive adrenergic stimulation and obstruction within the left ventricle's middle cavity during DSE could transiently trigger the occurrence of apical ballooning [103].

Flecainide, a class Ic antiarrhythmic drug that blocks sodium channels and slows conduction in cardiac tissue, was received by the patient at a recommended intravenous dose. This caused TCS to rise because of the decrease in 0 Cardiac contractility effect and/or sympathetic activation caused by Flecainide [13]. Anagrelide, a phosphodiesterase III inhibitor prescribed for essential thrombocythemia, is linked to mid-ventricular TCS in a 75-year-old woman. The adverse reaction to anagrelide therapy is determined to be a cumulative dose-dependent effect, leading to intensive inotropic stimulation and sympathetic hyperactivation in a vulnerable myocardium. This case highlights the possibility of anagrelide to induce an atypical Takotsubo-like syndrome [12].

Clonidine, an alpha-2 adrenergic agonist that impedes the CNS's sympathetic output, has been infrequently TCM association. It can regulate sympathetic activity and disturb the intricate equilibrium between sympathetic and parasympathetic tone might play a role in TCM development [16].

The DITC pathophysiology with immune-mediated inflammatory response has not been adequately explained. Moreover, it was hypothesized that the immune system contributes to the development. Atezolizumab, a monoclonal antibody intended the PD-L1, activates the immune system to combat cancer cells, leading to the release of pro-inflammatory cytokines like IL-6 and TNF-α. These cytokines could cause myocardial inflammation and damage. Additionally, the disclosure of stress-related catecholamines, including epinephrine and nor-epinephrine, potentially enhances the initiation of TCM. However, rare cases suggested a plausible connection between JAK inhibitors, including tofacitinib, and TCM development, possibly owing to their pro-thrombotic effects. The intricate interaction between the immune system, inflammatory cytokines, and catecholamines in TCM pathogenesis remained an active area of research [90-106].

Epinephrine bound to both beta 1-AR and beta 2-AR, exhibiting a higher affinity for beta 2-AR. Physiologically, it activated G-as, resulting in positive inotropy, lusitropy, and chronotropy. In this circumstance, elevated plasma levels activated G-ai, causing detrimental cardiac effects. Lyon *et al.* proposed an intriguing hypothesis about the cellular biology of beta-ARs and G-as/G-ai in TCM pathogenesis. They suggested that norepinephrine, released from sympathetic nerve endings, preferentially bound to the beta-AR on ventricular cardiomyocytes. This exerted positive inotropic and lusitropic effects *via* the g-as-adenyl cyclase-30, 50-cyclic adenosine monophosphate-protein kinase A pathway. Researchers have actively investigated the belief that intense stimulation leads to a switch in beta2-AR signaling from G-as to G-ai. This switch is referred to as stimulus trafficking or functional selectivity [107].

Acute management for drug-induced Takotsubo Cardiomyopathy (DITC) focuses on achieving and maintaining hemodynamic stability, which is of paramount importance. To achieve this, we implement various supportive measures, including administering supplemental oxygen, managing fluids carefully, and tracking pharmacological interventions such as vasopressors or inotropes, if needed, to ensure adequate cardiac output. Careful monitoring of vital signs, cardiac rhythm, and oxygen saturation is crucial, and continuous telemetry monitoring, along with regular assessment of cardiac biomarkers, should be employed. Additionally, appropriate management of pain, if present, is an essential aspect of supportive care for DITC patients. Paying careful attention to these aspects of acute management can contribute to better patient outcomes [81, 84, 108].

Levosimendan, a calcium sensitiser, has demonstrated beneficial effects in TTc patients and ought to as the first line therapy when switching from catecholamine inotropic drugs should be regarded as the first-line therapy when transitioning from catecholamine inotropic agents [109]. In acute heart failure management, guideline-directed therapy is crucial and typically involves a combination of angiotensin enzyme inhibitors or angiotensin II receptor blockers, beta-blockers, diuretics, and nitroglycerin [110]. Beta-blockers, in particular, may provide additional benefits by counteracting the excessive catecholamine effects and reducing the chance of cardiac rupture. Another important consideration in TTC patients is thrombus formation risk in left ventricular and subsequent systemic embolization. It must address these patients at risk who have severe left ventricular systolic dysfunction. In cases where a left ventricular thrombus is present, anticoagulation therapy should continue for a minimum of three months or until left ventricular function has normalized [111]. These management strategies aim to optimize cardiac function, reduce ventricular filling pressures, and prevent complications such as thrombus and embolism risk and cardiac rupture in TTC patients. Implementing guideline-directed therapy, considering levosimendan use, and carefully addressing thrombus formation are critical aspects of managing Takotsubo Cardiomyopathy effectively [112, 113]. The management of Angiotensin-converting enzyme inhibitors (ACEI) or Angiotensin II receptor blockers (ARB) in the International Takotsubo Registry improved survival at one-year follow-up and reduced the incidence of TTS recurrence [114, 115].

This review streamlines the process of identifying what might trigger TCM by providing an up-to-date list of relevant medications (Table **4**). The list proves to be an invaluable resource for identifying potential factors contributing to stimuli TCM, particularly in situations where emotional or stress-related triggers are not evident. This knowledge greatly aids healthcare professionals, including physicians and pharmacists, in efficiently managing at-risk patients and providing appropriate care for TCM.

One drawback of the systematic review is making use of one database (Medline/PubMed); however, this limitation was partially overcome by conducting a thorough reference search. Another limitation was the lack of definitive evidence of causality, as case reports were utilized. For instance, in cases where doctors treated anaphylaxis with epinephrine, there is a possibility that anaphylaxis contributed to the development of TCM. Some authors diagnosed TCM based solely on timing and the absence of other triggers, such as emotional or physical stress [10, 15-17]. Future research should focus on understanding the pathophysiology. It should also focus on the specific mechanisms by which certain drugs can trigger TCM. Given these limitations, caution should be exercised when interpreting and applying these findings.

## CONCLUSION

On the exhaustive evaluation of available information from the four reviews on DITM, a total of 192 cases of transient left ventricular ballooning syndrome have been documented from the year 1991 till present, out of which 75 drugs developed a stronger connection, either directly or indirectly of broken heart syndrome. Our study revealed that the drugs epinephrine, nor-epinephrine, and dobutamine can trigger TCM in older cardiac patients with decreased heart rate. These medications may be associated with this iatrogenic condition. Hence these collective findings further reinforce the SNS involved in triggering TCM. These results provide valuable insights for physicians and pharmacists in recognizing TCM cases. Yet, it is crucial to approach this information cautiously. Additional studies are necessary to establish definitive causality between drugs and TCM.

## Figures and Tables

**Table 1:** Combination of terms and comprehensive search of articles within the MEDLINE/PUBMED database.

**Combination of Terms**	**No. of Articles Retrieved**
Drug-induced apical ballooning syndrome	11
Drug-induced transient left ventricular ballooning syndrome	2
Drug-related broken heart syndrome	2
Iatrogenic TTC	4
Drug-induced TTC	13
Transient left ventricular ballooning AND drug induced	0
Stress cardiomyopathy AND iatrogenic	4
Tako-tsubo cardiomyopathy	32
Apical ballooning syndrome AND iatrogenic	4
Takotsubo cardiomyopathy AND drug-induced AND other possible combinations of searched term	0
Diseased Oriented	6
Total articles retrieved	78
Non-duplicate references retrieved	6

**Table 2:** A case report of takotsubo cardiomyopathy (Probably Drug Induce).

**Sl. No.**	**Age**	**Sex**	**Suspected Drug**	**Which Condition the Drug is Given**	**Dose**	**Duration**	**Adverse Events**
1.	63	W	Metoprolol [10]	Hypertension (suicide attempt)	12 g	First Day	Takotsubo Cardiomyopathy
2.	80	W	Atezolizumab [11]	Hepato-cellular carcinoma	1200 mg	Third Cycle	Takotsubo Cardiomyopathy
3.	75	W	Anagrelide [12]	Essential thrombocythemia	NA	NA	Takotsubo Cardiomyopathy
4.	30	W	Flecainide [13]	Focal atrial tachycardia	2 mg/Kg	1^st^ dose	Takotsubo Cardiomyopathy
5.	57	W	Tofacitinib [14]	Rheumatoid arthritis	10 mg	After 1 year	Takotsubo Cardiomyopathy
6.	14	W	Dobutamine [15]	DSE for Heart Transplantation	NA	1^st^ dose	Takotsubo Cardiomyopathy
7.	6 months old	M	Clonidine [16]	ADHD	NA	Untentional	Takotsubo cardiomyopathy

**Abbreviations:** Electrocardiogram (ECG), Takotsubo cardiomyopathy(TCM), Non-Specified (NS), Intravenous (IV).

**Table 3:** List of 7 suspected drugs that have the potential to induce TCM (4 from this review and the past reviews, and 3 from this review).

**Source**	**Suspected Drugs**	**Pharmacological Commentary**
Four drugs identified both in this update and in the past reviews	Flecainide [13]	Flecainide is a Class IC antiarrhythmic medication that acts by blocking sodium channels.
	Anagrelide [12]	It’s an anti-platelet and inhibits the maturation and production of megakaryocytes.
	Dobutamine [15]	Immediate metabolic precursor of Norepinephrine and epinephrine
	Metoprolol [10]	It is a sympathomimetic agent that selectively stimulates beta-1 adrenergic receptors.
Three drugs were identified in this update but not so in the 2022 review	Clonidine [16]	Alpha-2 adrenergic agonist lowers blood pressure by reducing sympathetic outflow from the brainstem.
	Tofacitinib [14]	JAK inhibitor that suppresses immune response and reduces inflammation in autoimmune diseases.
	Atezolizumab [11]	It’s a programmed death ligand 1 (PD-L1) inhibitor that enhances the immune response against cancer cells.

**Table 4:** List of the 75 identified drugs-induced TCM in 2011, 2015, 2022 and 2023 reviews.

**Year**	**Drug List**
2011 (n=18)	Cephalothin [17]
	Combretastatin [18]
	Dipyridamole [19]
	Ephedrine [20]*
	Ergonovine [21]*
	Levothyroxine [22]
	Lumiracoxib [23]
	Metoprolol [24]*
	Nortriptyline [25]*
	Oxymetazoline [26]*
	Anagrelide [27]*
	Atropine [28]*
	Potassium chloride [29]
	Dobutamine [30-32]*
	Duloxetine [33]*
	5-fluorouracil [34]
	Venlafaxine [35]*
	Epinephrine [36, 37]*
2015 (n = 37)	Amphetamine-Dextroamphetamine [38]*
	Bromocriptine [39]*
	Capecitabine [40]
	Ceftriaxone [41]
	Cytarabine [42]
	Cocaine [43]*
	Daunorubicin [44]*
	Dextromethorphan [45]*
	Desvenalafazine [46]*
	Digoxin [47]
	Flecainide [48]
	Human Immunoglobulin [49]
	Isocarboxazid & Phenethylamine [50]*
	Lidocaine [51]
	Lithium carbonate [52]
	Maprotilline [53]
	Meperidine [54]*
	Midodrine [55]*
	Milnacipran [56]*
	Morphine [57]*
	Nandrolone & Stanozolol [58]
	Neostigmine [59]*
	Oxaliplatin [60]
	Rituximab [61]
	Sunnicylcholine [62]
	Sulprostone [63]
	Sunitinib [64]
	Phentermine [50]
	Pseudoephedrine [65]*
	Terlipressin [66]
	Trastuzumab [67]
	Zolmitriptan [68, 69]
	Norepinephrine [70-72]*
	Phenylephrine [73, 74]*
2022 (n = 15)	Albuterol [75]*
	Axitinib [76]
	Buprenorphine [77, 78]*
	Bevacizumab [79, 80]
	Dopamine [81]*
	Entacapone [82]*
	Ergotamine [83]*
	Ipratropium bromide [84]*
	Intraleukin-2 [85]
	Methylprednisolone [86]
	Polidocanol [87]
	Rosuvastatin [88]
	Sodium tetradecyl sulphate [89]
2023 (n = 3)	Clonidine [16]*
	Tofacitinib [14]
	Atezolizumab [11]

**Note:** *represents drug-associated with direct or indirect excessive stimulation of the SNS.

## Data Availability

All the data-supportive information is provided within the article.

## LIST OF ABBREVIATIONS

Beta 2-AR: Beta 2 Adrenergic Receptor
CNS: Central Nervous System
DITC: Drug Induced Takotsubo Cardiomyopathy
DSC: Dopamine Stress Echocardiogram
G-ai: G Protein-coupled Inhibitory Receptor
G-as: G Protein-coupled Stimulus Receptor
IL-6: Interleukin 6
LVOTO: Left Ventricular Outflow Tract Obstruction
PD-L1: Programmed Death Ligand 1
SNS: Sympathetic Nervous System
TAD: Takotsubo Cardiomyopathy
TNF-α: Tumor Necrosis Factor Alpha
TTS: Takotsubo Syndrome

## STANDARDS OF REPORTING

PRISMA guidelines and methodology were followed.

PRISMA checklist is available on the publisher’s website.

## SUPPLEMENTARY MATERIAL

PRISMA checklist and flowchart are available as supplementary material on the publisher’s website along with the published article. Supplementary material is available on the publisher’s website along with the published article.
